# Clinical experience with hepatorenal tyrosinemia from a single Egyptian center

**DOI:** 10.1371/journal.pone.0268017

**Published:** 2022-05-10

**Authors:** Hanaa El-Karaksy, Hala Mohsen Abdullatif, Carolyne Morcos Ghobrial, Engy Adel Mogahed, Noha Adel Yasin, Noha Talal, Mohamed Rashed

**Affiliations:** 1 Department of Pediatrics, Cairo University, Cairo, Egypt; 2 Pharmagene Specialized Analytical Services, Cairo, Egypt; Sohag University Faculty of Medicine, EGYPT

## Abstract

Although very recently, in Egypt, sick newborn screening has included screening for hepatorenal tyrosinemia, yet, it is not yet included in nationwide neonatal screening and hence diagnosis may be delayed. The aim of this study was to analyze data of all cases presenting with hepatorenal tyrosinemia to the Pediatric Hepatology Unit, Cairo University, Egypt from 2006 to 2019. Data were retrieved from patients’ files including age of onset of symptoms, clinical signs, blood counts, liver functions, serum phosphorous, alpha-fetoprotein, succinylacetone and abdominal ultrasound. During this period, 76 patients were diagnosed with hepatorenal tyrosinemia if succinylacetone in dry blood spot was elevated above 1 μmol/L. These 76 cases came from 70 families; consanguinity was reported in 61 families. In our cohort we reported 30 affected siblings with a similar clinical presentation, who died undiagnosed. Presentation was acute in 26%, subacute in 30% and chronic in 43%. Abdominal distention was the commonest presenting symptom (52.6%). Coagulopathy was the commonest derangement in liver functions; hyperbilirubinemia and raised transaminases were less common. Ultrasound findings included hepatic focal lesions in 47% and enlarged echogenic kidneys in 39% and 45.3% respectively. Only 20 children were treated with Nitisinone because of unavailability and high costs; seven out of them underwent liver transplantation. In conclusion, although hepatorenal tyrosinemia is a rare inborn error of metabolism, in a large population country with high rate of consanguinity; this disease is not uncommonly diagnosed. The current treatment is not readily available because of the costs in a resource-limited country. Neonatal screening and subsidization of the costly medication need to be considered.

## Introduction

Tyrosinemia type I (hepatorenal tyrosinemia, HT-1) is an autosomal recessive condition (OMIM 276700) resulting in hepatic failure with comorbidities involving the renal and neurologic systems [1, 2]. It is characterized by chronic liver disease that progresses into cirrhosis and is commonly associated with malignant transformation [3]. Renal involvement is characterized by renal tubular acidosis and hypophosphatemic rickets [4]. According to age of onset and clinical symptoms it is classified into acute, subacute and chronic forms [5–7].

Most literature on HT1 comes from Western countries [8–10], particularly the province of Quebec, Canada [4, 11]. Literature about diagnosis and management of HT1 from resource-limited countries is scarce [12–14], including Egypt.

In Egypt, the diagnostic test for HT1 became available in 2006. Despite the high rate of consanguinity among Egyptians [15] and relatively large family sizes, this rare metabolic disorder is still under-diagnosed. In our busy tertiary care center, that receives over 300 new pediatric liver disease cases per year, we have been receiving new cases with HT1 over the last 14 years, however, unavailability of the costly medication 2-(2-nitro-4-trifluoro-methylbenzoyl)-1,3-cyclohexanedione (NTBC) is an obstacle in improving the dismal outcome of untreated cases. Although very recently, in Egypt, sick newborn screening has included screening for HT1, yet, it is not yet included in nationwide neonatal screening and hence diagnosis may be delayed.

We aimed to analyze the data of all patients diagnosed with HT1 presenting to the Pediatric Hepatology Unit at Cairo University Pediatric Hospital, Egypt over the last 14 years from 2006 through 2019 in an attempt to highlight the common clinical presentations and speculate on causes for delayed diagnosis. According to our knowledge, this is the first report on this big number of patients with hepatorenal tyrosinemia from a single center in Egypt.

## Materials and methods

This retrospective data-based observational study included all patients diagnosed with HT1 from 2006 till the end of 2019. The study protocol was approved by the Institutional Review Board and Ethical committee. Informed consent was waivered because the analysis used anonymous data obtained from patients’ files.

Diagnosis of HT1 was achieved by quantification of succinylacetone in dried blood spots [16]. Positive result was considered if succinylacetone was >1 μmol/L. Succinylacetone (SA; 4,6-dioxoheptanoic acid) was determined by liquid chromatography tandem mass spectrometry (LC-MS/MS). Briefly, SA was extracted from dried blood spots or from urine in acidified medium containing isotope labeled SA (^13^C_4_). The mixtures were heated to 100°C to convert any succinylacetoacetate to SA. After cooling, the mixtures were treated with excess sodium chloride and then extracted with ethyl acetate. The extracts were then centrifuged, evaporated to dryness with nitrogen. The dried residues were then treated with butanolic HCl and heated at 65°C for 15 min to make the butyl esters. Excess butanolic HCl was removed by evaporation to dryness with nitrogen. The residues were reconstituted with 100 ml of 2.5mM of dansylhydrazine in acetonitrile and 10 ml trifluoroacetic (2.5%, v/v) and allowed to stand at room temperature for 1 h in the dark and 10 ml were analyzed by LC-MS/MS using a C8 Waters Symmetry column. The transitions for the butylated and dansylated SA and internal standard were m/z 462 to 170 and m/z 466 to 170, respectively. Under these conditions the analytes eluted around 2.5 min and total run time was 5 min per sample. Linear calibration curves for blood spots were in the range of 1–100 μM and for urine were 0.025 to 30 μM.

Analysis of amino acids from dried blood spots: The same dried blood spots (DBS) used for determination of succinylacetone were also analyzed for amino acids such as methionine, tyrosine and phenylalanine among others as well as acylcarnitines. The method used for this analysis was described by Rashed et al. [17] and Turgeon et al. [18]. In brief we extracted 1/8-inch DBS punches with methanol containing amino acids and acylcarnitines stable isotope-labeled internal standards obtained from Cambridge Isotopes (MA, USA). The methanolic extracts were dried and derivatized with butanolic HCl at 65°C for 15 minutes. Excess butanol was evaporated and the dried residues were reconstituted in 80% acetonitrile. These solutions were analyzed for amino acids and acylcarnitines by flow injection electrospray tandem mass spectrometry on an API3200 triple quadrupole system (Sciex, USA).

Testing for succinylacetone was performed for infants and children with evidence of liver disease (hepatomegaly/hepatosplenomegaly) and any of the following: 1) clinical and/or radiological evidence of rickets; 2) coagulopathy (INR>1.5); 3) abdominal ultrasound showing heterogenous liver parenchyma, focal hepatic lesions, increased renal size or echogenicity; and 4) elevated alpha fetoprotein for age.

Data were extracted from paper files by HE and double checked by EAM. The following data were retrospectively retrieved from patients’ files.

1. Date of birth, sex, date of first presenting symptom, and date of presentation to our unit and diagnosis; delay in diagnosis from first symptom to presentation to our unit was calculated in months. According to age at first presenting symptom, cases were classified into acute presentation between 0–2 months of age, subacute presentation between 2–6 months and chronic presentation older than 6 months.
2. Presenting symptoms: abdominal distention, jaundice, bleeding, failure to thrive, delayed motor milestones, edema, fever, fractures, polyuria, polydipsia, dehydration and infections.
3. Parental consanguinity and previously affected sibs with a similar condition
4. Physical signs: jaundice, hepatomegaly, splenomegaly, ascites, rickets, edema and bruising.
5. Laboratory tests: hemoglobin, platelet counts, full liver functions (total and direct serum bilirubin, alanine aminotransferase [ALT], aspartate aminotransferase [AST], alkaline phosphatase [AP], serum albumin and international normalized ratio [INR]), serum phosphorus, serum alpha fetoprotein, succinylacetone, urine analysis and tandem mass spectrometry when available.
6. Abdominal ultrasound data with special stress on homogenicity and echogenicity of hepatic parenchyma and presence of focal hepatic lesions, the kidney size and echogenicity and presence of ascites.
7. Data on the cases that received treatment whether medical treatment using NTBC or liver transplantation were included.

### Statistical analysis

Data were coded and entered using the statistical package SPSS (Statistical Package for the Social Sciences) version 25. Data were summarized using mean, standard deviation (SD), median and interquartile range (IQR), minimum and maximum in quantitative data and using frequency and percentage for categorical data. Comparisons between quantitative variables were done using the independent t-test for parametric data and Mann-Whitney test for non-parametric data. P-value less than 0.05 was considered statistically significant.

## Results

**Fig 1:**
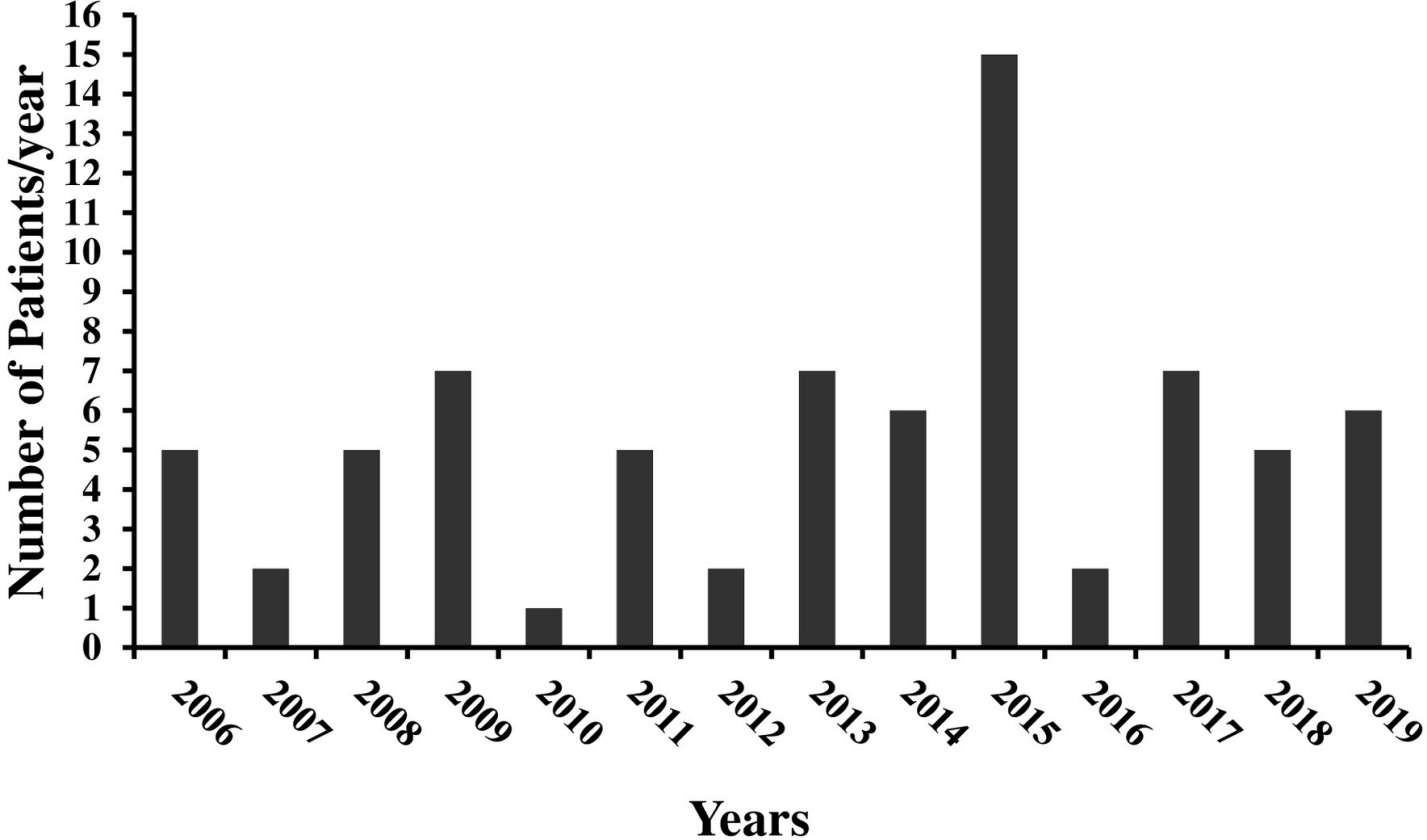
Frequency of cases of hepatorenal tyrosinemia diagnosed over the years of the study.

The study included all cases diagnosed with HT1 at the Pediatric Hepatology Unit, Cairo University Pediatric Hospital, Cairo, Egypt from 2006 through 2019. Over these 14 years, we diagnosed 76 cases with HT1; 43 (56%) were females. In the first 7 years, from 2006–2012, we diagnosed 28 cases and from 2013–2019 we diagnosed 48 cases. The rate of presenting cases in the 1^st^ 7 years ranged between 1 and 8 cases/year with a mean of 4 cases/year, while in the following 7 years the rate was 6.6 cases/year, ranging between 2 and 15 cases/year **(Fig 1).**

According to the date of birth, those born between 2004 and 2010, the median (IQR) delay to diagnosis was 7 (1–14) months, while for those born between 2011 and 2017 the median (IQR) delay was 4 (1–8) months; however, the difference was not statistically significant (p = 0.98).

Our 76 cases came from 70 families; as our cohort included 6 pairs of brothers and sisters from 6 families. Consanguinity was reported in 61 families. To note, 2 non-consanguineous parents had 2 affected children. Thirty affected siblings with a similar clinical presentation, who died undiagnosed, were reported in our cohort. Twenty-one families had 2 affected sibs, 5 had 3 affected sibs, 3 had 4 affected sibs and 1 family had 5 affected sibs.

The main presenting symptoms were: abdominal distention in 40 (52.6%), accidentally discovered hepatomegaly, splenomegaly or hepatosplenomegaly in 15 (19.7%), bleeding, most frequently epistaxis or discovered during pre-operative preparation for minor surgery in 11 (14.5%), jaundice in 7 (9.2%), delayed motor development and rickets in 6 (7.9%), recurrent fever in 6 (7.9%), diarrhea and/or vomiting in 6 (7.9%), accidently discovered hepatic focal lesions by ultrasound, failure to thrive and edema each in 5 (6.6%). Polyuria and polydipsia were reported in 3 (4%) and repeated dehydration in 2 (2.6%). One case presented after an episode of paralysis that necessitated mechanical ventilation and was diagnosed as Guillian Barre-like syndrome; her older brother died of a similar condition. Six cases were screened for HT1 using succinylacetone test because the family had a previously affected sibling.

On examination, 70 (92%) had organomegaly (hepatomegaly, splenomegaly, or hepatosplenomegaly), 34 (44.7%) had rickets (one with fractures), jaundice in 17 (22%), ascites in 10 (13%), generalized edema in 4 (5.3%) and fever in 4 (5.3%). The patient with an episode of paralysis, diagnosed as Guillian Barre-like, had rickets and no organomegaly.

According to age at presentation [6], 20 cases had an acute presentation (26.3%), 23 subacute (30.3%) and 33 had chronic presentation (43.4%). The presenting symptoms of the 3 groups are shown in **Table 1.** No statistically significant differences were noted between the 3 groups, except for jaundice and coagulopathy, which were least reported in the chronic form (p = 0.013 and p = 0.033 respectively).

**Table 1:** Presenting symptoms in 76 hepatorenal tyrosinemia cases according to age of onset of symptoms.

Symptom	Acute	Subacute	Chronic	p-value
	N = 20	N = 23	N = 33	
	N (%)	N (%)	N (%)	
Jaundice	4 (20)	9 (39.1)	3 (9.1)	0.033*
Hepatomegaly	18 (90)	20 (87)	27 (81.8)	0.942
Splenomegaly	17 (85)	16 (69.6)	23 (69.7)	0.485
Ascites	4 (20)	2 (8.7)	4 (12)	0.552
Bleeding	4 (20)	9 (39.1)	2 (6)	0.013*
Rickets	9 (45)	9 (39.1)	16 (48.5)	0.658
Edema	0 (0)	1 (4.3)	3 (9.1)	0.317
Fever	1 (5)	2 (8.7)	0 (0)	0.269
Failure to thrive	1 (5)	0 (0)	0 (0)	0.254
Guillian Barre-like	0 (0)	0 (0)	1 (3)	0.495

*P-value is significant

Anemia was present in 78% of the cases and thrombocytopenia in 48.7% **(Table 2).**

**Table 2:** Hematological findings in 76 hepatorenal tyrosinemia cases.

Variable	
**Hemoglobin (gm/dl)**	
** Range**	5.5–13.2
** Median (IQR)**	9.25 (8.23–10.2)
**Anemia; N (%)**	59 (77.6)
**NA; N (%)**	7 (9.2)
**Platelets (/mm** ^**3**^ **)**	
** Range**	37,000–503,000
** Median (IQR)**	143 (97.5–193.5)
**Thrombocytopenia; N (%)**	37 (48.7)
**100,000–150,000 (/mm**^**3**^**)**	19 (25)
**50,000–100,000 (/mm**^**3**^**)**	15 (19.7)
**<50,000/mm**^**3**^	3 (3.9)
**NA; N (%)**	9 (11.8)

IQR = interquartile range; NA = not available

Results of liver function tests are shown in **Table 3.** Elevated total serum bilirubin (>1.2 mg/dl) was encountered in 41 cases, elevated direct serum bilirubin (>0.2 mg/dl) was present in 56 cases, AST was elevated above the upper limit of normal (40 IU/L) in 66 cases and ALT was elevated above upper limit of normal (40 IU/L) in 21 cases. Serum albumin was reduced in 38 cases (<3.5 gm/dl) and coagulation was deranged in 62 cases (INR >1.3).

**Table 3:** Liver function tests of 76 hepatorenal tyrosinemia cases.

Variable	
**Total serum bilirubin (mg/dl)**	
**Range**	0.16–6.3
**Median (IQR)**	1.9 (1–2.8)
**Raised (> 1.2 mg/dl); N (%)**	41 (54)
**NA; N (%)**	12 (15.8)
**Direct serum bilirubin (mg/dl)**	
**Range**	0.1–3.5
**Median (IQR)**	0.7 (0.3–1.3)
**Raised (> 0.2 mg/dl); N (%)**	56 (73.7)
**NA; N (%)**	10 (13.2)
**AST (IU/L)**	
**Range**	20–241
**Median (IQR)**	84 (61–119)
**Elevated >40 IU/L; N (%)**	66 (86.8)
**40–80 IU/L; N (%)**	32 (42.1)
**80–120 IU/L; N (%)**	18 (23.7)
**120–160 IU/L; N (%)**	7 (9.2)
**>160 IU/L; N (%)**	9 (11.8)
**NA; N (%)**	7 (9.2)
**ALT (IU/L)**	
**Range**	10–84
**Median (IQR)**	33 (23–44)
**Elevated >40 IU/L**	21 (27.6)
**40–80 IU/L; N (%)**	19 (25)
**80–120 IU/L; N (%)**	2 (2.6)
**NA; N (%)**	5 (6.6)
**Serum albumin(gm/dl)**	
**Range**	2–4.7
**Median (IQR)**	3.2 (2.7–3.85)
**Decreased (<3.5 gm/dl); N (%)**	38 (50)
**3–3.5 gm/dl**	16 (21.1)
**2.5–3 gm/dl**	12 (15.8)
**2–2.5 gm/dl**	10 (13.2)
**NA; N (%)**	10 (13.2)
**INR**	
**Range**	1–5.7
**Median (IQR)**	2.2 (1.7–3)
**Deranged (≥1.3); N (%)**	62 (81.6)
**INR> 2**	39 (51.3)
**NA; N (%)**	14 (18.4)

ALT = alanine aminotransferase; AST = aspartate aminotransferase; INR = international normalized ratio; IQR = interquartile range; NA = not available.

**Fig 2:**
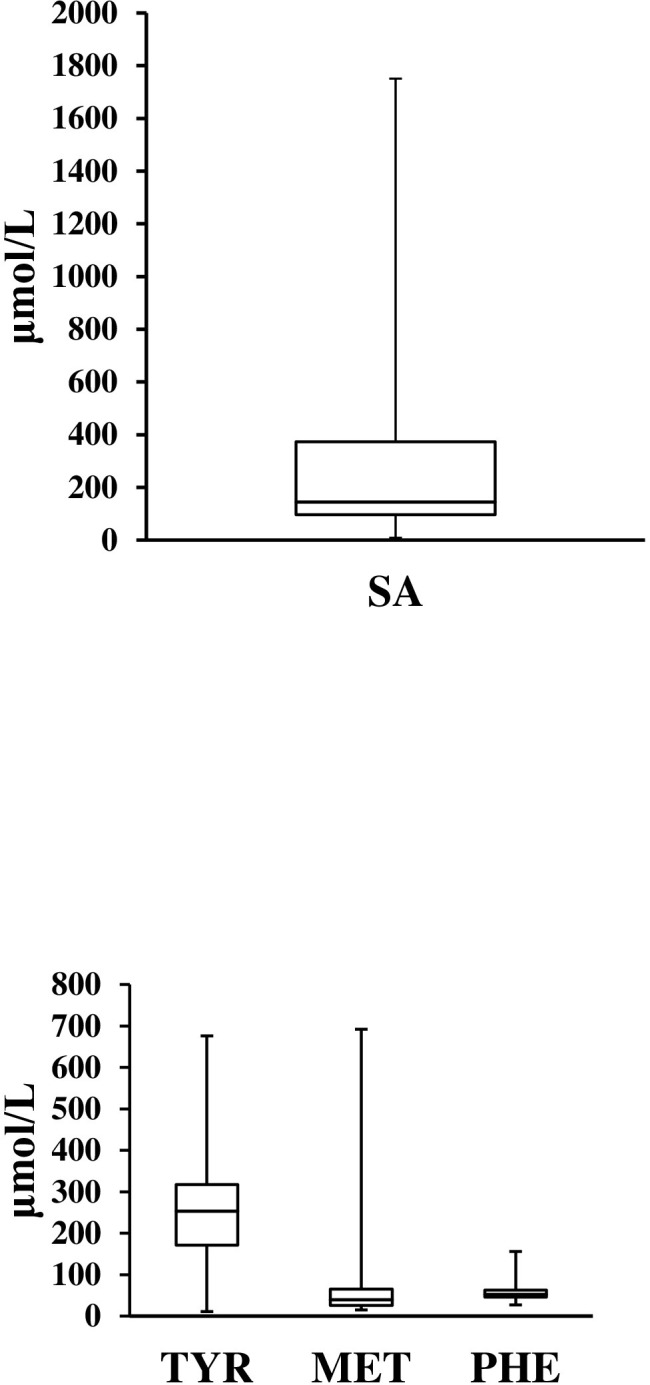
The median levels of succinylacetone, tyrosine, methionine and phenylalanine (in μmol/L) in patients with hepatorenal tyrosinemia. METH = methionine; PHEN, phenylalanine; SA = succinylacetone; TYR = tyrosine.

Reduced serum phosphorous (<4.3 mg/dl) was noted in 72%. Alpha fetoprotein was elevated in all 66 tested cases. Succinylacetone, the diagnostic test was positive in all cases ranging from 8.5 μmol/L to 1751 μmol/L with a median (IQR) of 144.5 (96.25–373) **(Table 4).** Results of tandem mass spectrometry are shown in **Table 4 and Fig 2A and 2B.**

**Table 4:** Relevant biochemical tests of 76 hepatorenal tyrosinemia cases.

Variable	
**Alkaline phosphatase (IU/L)**	
**Range**	190–7498
**Median (IQR)**	1488 (861–2325)
**Elevated; N (%)**	68 (89.5)
**NA; N (%)**	6 (7.9)
**Serum Phosphorous (mg/dl)**	
**Range**	1–5.7
**Median (IQR)**	2.1 (1.4–2.9)
**Decreased (<4.3 mg/dl); N (%)**	55 (72.4)
**NA; N (%)**	17 (22.4)
**Alpha fetoprotein (ng/ml)**	
**Range:**	94–97631
**Median (IQR)**	18927.5 (9775–41498.25)
**Elevated; N (%)**	66 (86.8)
**NA; N (%)**	10 (13.2)
**Succinylacetone (μmol/L)**	
**Range**	8.5–1751
**Median (IQR)**	144.5 (96.75–373)
**Elevated; N (%)**	76 (100)
**Tyrosine (μmol/L)**	
**Range:**	10.9–676
**Median (IQR)**	253 (171–317)
**Elevated; N (%)**	19 (70.3)
**NA; N (%)**	49 (64.4)
**Methionine (μmol/L)**	
**Range:**	15–692
**Median (IQR)**	39.4 (25.92–64.75)
**Elevated; N (%)**	10 (38.5)
**NA; N (%)**	50 (65.8)
**Phenylalanine (μmol/L)**	
**Range:**	26.9–156
**Median (IQR)**	52.3 (46.22–62.71)
**Elevated; N (%)**	1 (8.3)
**NA; N (%)**	64 (84.2)
**Urinalysis**	
**Glucosuria; N (%)**	10 (13.2)
**Albuminuria; N (%)**	3 (3.9)
**Alkaline pH; N (%)**	6 (7.9)
**NA; N (%)**	51 (67.1)

Abdominal ultrasound was available in 64 files. Focal hepatic lesions were detected in 30 cases; the texture was heterogenous in 23, bright in 7, coarse in 3 and normal in 3. The kidneys were echogenic in 29 cases and there was nephromegaly in 25. The kidneys were reported normal in 17 cases. Ascites was detected by ultrasound in 4 cases **(Table 5).**

**Table 5:** Abdominal ultrasound findings in 64 cases of hepatorenal tyrosinemia.

Variable	N (%)
**Liver**	
**Focal hepatic lesions**	30 (47)
**Heterogenous**	23 (36)
**Bright**	7 (11)
**Coarse**	3 (4.7)
**Normal**	3 (4.7)
**Kidney**	
**Echogenic**	29 (45.3)
**Enlarged**	25 (39)
**Normal**	17 (26.6)
**Ascites**	4 (6.3)

## Discussion

For the last 2 decades, Egypt has implemented a neonatal screening program for congenital hypothyroidism and few years ago phenylketonuria was added to the program. More recently, sick newborn screening in neonatal intensive care units, included screening for HT1. However, nationwide neonatal screening for HT1 has not been implemented yet. Local data need to be addressed in order to call for action for this treatable inborn error of metabolism. The increase in number of cases presenting to our unit over time may indicate some increased awareness among physicians, particularly because some publications about this rare condition came from Egypt [12, 13, 19]. Our current cohort includes our first 22 cases we previously reported in 2011 [13]. Besides the 76 diagnosed cases with HT1 in the present study, 30 brothers and sisters were reported to die undiagnosed, which reflects the importance of raising more awareness in the medical community of this rare inherited disorder. Still, this number of HT1 cases underestimates the problem in Egypt with a population exceeding 100 million and a high rate of consanguinity. If we assume that the frequency of HT1 is similar to the reported worldwide incidence of 1:100,000 [20], then at least 20 cases are born annually. Our center is probably receiving most of the cases countywide. Similar results were reported from Jordan [21], where no nationwide neonatal screening is applied for HT1.

### Is there a delay in diagnosis? Why?

Although the interval from first symptom to diagnosis came down from a median (IQR) of 7 (1–14) months for those born from 2004–2010 to 4 (1–8) months for those born from 2011–2017, yet the difference was not statistically significant.

Does a normal ALT add to the delay in diagnosis? It is evident in our cohort that almost two thirds of the cases have normal ALT, and those with elevated ALT showed a mild increase mostly within 2–3 folds the upper limit of normal. Fumaryl acetoacetate, accumulating as the result of the metabolic block resulting from fumaryl acetoacetate hydralase deficiency, induces genome instability by activation of the ERK pathway [20]. It also induces cell cycle arrest and apoptosis through glutathione depletion [22, 23]. Because hepatocytes undergo apoptosis, the ALT is not expected to rise as high as in other liver disease characterized by marked ALT elevations. Moreover, jaundice was not a prominent presenting symptom among our cohort, only 11 parents came complaining that their infants were jaundiced, although by clinical examination 16 patients had jaundice. Serum total and direct bilirubin levels were usually too low to be observed by parents, although hepatorenal tyrosinemia is always included among causes of cholestatic disorders of infancy [24–26]. Hyperbilirubinemia is not helpful in the diagnosis of hepatorenal tyrosinemia. Its presence may point to another liver problem [2]. The mainstay in disturbed liver functions is coagulopathy. Bleeding and impaired coagulation were more noted as a symptom and as a lab finding than jaundice or elevated transaminases. Half of the cases had an INR above 2, not correctable with vitamin K, which explains the dismal outcome of these infants without treatment. Isolated coagulopathy [2] has to raise the suspicion towards this rare rapidly progressive disorder.

The patient whose presentation was by acute peripheral neuropathy, porphyria-like picture, came to medical notice because she had a previously affected brother who died. Porphyria-like crisis is caused by accumulation of delta-aminolaevulinic acid because of the inhibitory effect of succinylacetone on porphobilinogen synthase [27]. This girl outlived the event because treatment of the paralytic episode is intravenous glucose, control of pain, hypertension and hyponatremia [2]. NTBC is used to prevent further crises [28].

One of our families had: a) one older daughter that died undiagnosed with HT1; b) a second daughter diagnosed with HT1 who received NTBC for one year and was later successfully transplanted in 2009 and is surviving and doing well; c) a third affected daughter in 2017, 8 years after her sister received liver transplantation. Because of parental denial, diagnosis was delayed until 18 months of age. The parents were arguing that they never mentioned their 2^nd^ daughter’s condition to their local physician because the 2 presentations were different; the 2 older sisters presented with abdominal distention and were known to have hepatosplenomegaly, while their last daughter had motor delay and has been treated for rickets.

Alkaline phosphatase is usually markedly elevated in HT1, probably secondary to bone disease and hypophosphatemic rickets. Normal AP in 2 of our cases, with low serum phosphorous, was not justifiable except if we could prove that both were zinc deficient; AP is a zinc-dependent enzyme [29]. Zinc status was not tested in both cases. Although bone disease, and even fractures, was the presenting symptom in 6 cases, it was evident clinically in 30 patients on examination. Even the case that presented with neurological presentation, she had clinical and radiological evidence of rickets, with increased AP and low serum phosphorous, despite normal abdominal examination, normal abdominal ultrasound and normal liver functions and coagulation profile.

Alpha fetoprotein was uniformly elevated in all tested cases. It was elevated beyond age limits. Alpha fetoprotein has to be interpreted in young infants as it is the remnant of alpha fetoprotein, the major circulating protein in the fetus [30, 31]. Timely early diagnosis of HT1 and appropriate medical treatment with NTBC prevents the development of hepatocellular carcinoma [9]. Our patients who received NTBC showed a rapid decline in alpha fetoprotein as previously reported [12, 13].

Treatment with NTBC is not regularly available in Egypt. This orphan drug is too costly. In our center we had 2 opportunities to treat some patients provided compassionately by some benevolent donors. What was universally noted in all treated patients is the rapid decline in alpha fetoprotein and normalization of coagulopathy. Our first experience was published in 2011 [12, 13]. Our recent experience in the past few years with NTBC included another 10 patients. Five underwent successful liver transplantation after a variable period of NTBC treatment ranging from 10–14 months. These children responded well to NTBC, with growth spurts, healing of rickets, disappearance of focal hepatic lesions and steep fall in alpha fetoprotein. Unfortunately, they could not be maintained on treatment, because of financial issues, we recommended liver transplantation while in healthy state. All patients who received a liver transplant, after a period of NTBC therapy, between 2008 and 2020 are surviving till the time of writing this manuscript. Despite the aforementioned good response to NTBC, because treatment was not started in the first few months of life, the explanted livers were completely cirrhotic. The remaining 5 children have been provided for by different compassionate providers, hoping that in the near future the medication could be provided by the health insurance. It is to be noted that all transplanted patients continued to show low levels of succinylacetone in blood and urine despite adequate growth, normal urinalysis, serum phosphorous, serum creatinine, creatinine clearance and renal ultrasound. None of our patients received NTBC post-transplantation.

Our experience with liver transplantation for HT1 included 10 children; 9 following treatment with NTBC, and one without prior medical treatment. All 9 cases transplanted after a variable period of NTBC therapy, not less than 10 months, had a successful transplantation with survival ranging from 5 months to 11 years. Those who were treated medically pre-transplant survived the immediate post-operative period and till the time of writing the manuscript are all surviving. On the other hand, the case that did not receive treatment pre-transplant, died in the immediate post-operative period. In countries where NTBC is not readily available, because of cost or unavailability, is it wise to ensure at least several months of treatment to obtain better results with liver transplantation? A question that has to be discussed with experts. It is to be noted that all explanted livers, from HT1 patients who were transplanted in our center, were cirrhotic, even the case that started treatment as early as 6 months of life. This may call for neonatal screening and early implementation of treatment before silent cirrhosis sets in. Liver transplantation still has a role in cases with liver failure, unresponsive to NTBC, malignancy or where NTBC is not available [32, 33]. In the 15-year study on long-term safety and efficacy of NTBC, liver transplantation occurrence was more frequent if treatment with NTBC was not introduced early in life [34]. Although the quality of life after successful liver transplantation is usually good, however, there are the risk of surgery and immunosuppression [8]. Although transplanted patients continued to show positive succinylacetone in blood and urine in low quantities, NTBC was not given to any of them.

Long-term use of NTBC has to be associated with a low tyrosine diet. This restrictive diet requires adherence, as non-adherence leads to hypertyrosinemia that may lead to cognitive dysfunction [35], in addition to deposition of corneal crystals causing photophobia.

### Study limitations

Our study included data collected in retrospect from patients’ files, however because this considerable number of patients were diagnosed in a single center, we thought it was worth publication because of deficient data on this rare disease from resource limited countries. Most of our cases were not treated because of the high costs of the medication and its unavailability. We did not confirm the diagnosis of our cases by genetic analysis, except in a limited number of cases [16]. Genetic confirmation was recommended by a group of experts from US and Canada with a 100% agreement and evidence quality D [2]. We depended on succinylacetone assays for diagnosis although Blackburn et al. [36], reported a family with 3 affected sibs with cirrhosis and hepatosplenomegaly who had a novel mutation in the *FAH* gene who had no elevated tyrosine or succinylacetone. Succinylacetone is a pathognomonic marker of HT1 as it is only generated from maleyl- and fumarylacetoacetic acids when FAH is deficient. Methods have been developed overtime [37–40] making results more objective. A molecular genetic approach to NBS for HT1 has not yet been suggested and it is unlikely to be cost effective because, even in populations where a specific mutation may be prevalent, full gene sequencing and deletion testing would be required to ensure all patients are identified [41]. It is worth mentioning that any insignificant results reported might be attributed to the small number of patients.

## Conclusion

Egypt is a limited resource country in which the frequency of HT1 may be under-estimated. Abdominal distention, and not jaundice, is the salient presenting feature. Hyperbilirubinemia and increased transaminases are uncommonly encountered. Coagulopathy is very common and alpha-fetoprotein is uniformly elevated. Hypophosphatemic rickets is common. NTBC is still not available and very costly. Liver transplantation following a period of treatment with NTBC gives better results that transplantation without pre-treatment with NTBC. Sick newborn screening is being implemented nowadays and it is recommended to include assay for succinylacetone to avoid missing HT1 cases with normal tyrosine at birth. Nationwide neonatal screening for HT1 is still unavailable. The current information may raise the awareness about this treatable metabolic error and may be a call for action for these patients with dismal outcome if left untreated.

## Supporting information

S1 Data (XLSX)
